# Is waist-to-height ratio the best predictive indicator of hypertension incidence? A cohort study

**DOI:** 10.1186/s12889-018-5177-3

**Published:** 2018-02-26

**Authors:** Ana Carolina Rezende, Ludimila Garcia Souza, Thiago Veiga Jardim, Naiana Borges Perillo, Ymara Cássia Luciana Araújo, Samanta Garcia de Souza, Ana Luiza Lima Sousa, Humberto Graner Moreira, Weimar Kunz Sebba Barroso de Souza, Maria do Rosário Gondim Peixoto, Paulo César Brandão Veiga Jardim

**Affiliations:** 1 Nutrition and Health Post Graduation Program, Nutrition School, Federal University of Goiás, Rua 227, Quadra 68 s/n. Setor Leste Universitário, Goiânia, Goiás 74.605-080 Brazil; 2 Hypertension League, Federal University of Goiás, Avenida Primeira Avenida, s/n, Setor Leste Universitário, Goiânia, Goiás 74605-020 Brazil; 3 Goiânia, Brazil

**Keywords:** Waist-to-height ratio, Waist circumference, Body mass index, Obesity, Hypertension, Cohort studies

## Abstract

**Background:**

The best anthropometric indicator to verify the association between obesity and hypertension (HTN) has not been established. We conducted this study to evaluate and compare the discriminatory power of waist-to-height ratio (WHtR) in relation to body mass index (BMI) and waist circumference (WC) in predicting HTN after 13 years of follow-up.

**Methods:**

This study was an observational prospective cohort study performed in the city of Firminópolis, in Brazilian’s midwest. The cohort baseline (phase 1) was initiated in 2002 with the evaluation of a representative sample of the normotensive population (≥ 18 years of age). The incidence of HTN was evaluated as the outcome (phase 2). Sociodemographic, dietary and lifestyle variables were used to adjust proportional hazards models and evaluate risk of HTN according to anthropometric indices. The areas under the receiver operating characteristic (ROC) curves were used to compare the predictive capacity of these indices. The best HTN predictor cut-offs were obtained based on sensitivity and specificity.

**Results:**

A total of 471 patients with a mean age of 38.9 ± 12.3 years were included in phase 1. The mean follow-up was 13.2 years, and 207 subjects developed HTN. BMI, WC and WHtR were associated with risk of HTN incidence and had similar power in predicting the disease. However, the associations were only significant for women. The cut-off points with a better HTN predictive capacity were in agreement with current recommendations, except for the WC in men.

**Conclusions:**

The results suggest that both overall obesity (BMI) and central obesity (WC and WHtR) anthropometric indicators can be used in this population to evaluate the risk of developing hypertension.

**Electronic supplementary material:**

The online version of this article (10.1186/s12889-018-5177-3) contains supplementary material, which is available to authorized users.

## Background

Hypertension (HTN) is an important health concern in many countries. Global HTN prevalence in individuals ≥18 years was approximately 22% in 2014 [1], and at least 45% of coronary heart disease deaths and 51% of stroke deaths were attributable to HTN [2]. In 2010, 9.4 million deaths worldwide were associated to HTN [1].

Excessive body fat is one of the main risk factors associated to HTN. Risk estimates, based on population studies, indicate that 75% of HTN diagnoses are attributable to obesity [3, 4]. Central obesity is suggested to be even more strongly associated with blood pressure values than total adiposity [5].

Epidemiological studies have shown a relationship between HTN and anthropometric indicators that reflect excess body fat [6–8]. The main indicators used are the body mass index (BMI) and waist circumference (WC) [9–11]. Although BMI and WC are widely used in clinical practice and have been applied in several studies, there are still some limitations with these indicators.

For example, BMI is only related to total body fat and makes no distinction between the fat-free mass and fat mass. Therefore, the use of the BMI does not reflect differences in body composition according to the individual’s gender, age and ethnicity [10, 12]. The main limitation of the WC is that the use of this measure in isolation may underestimate or overestimate the health risks for tall and short individuals with similar WCs [13], because there is relatively strong evidence of an inverse association between height and health risks [14, 15]. Studies show that shorter individuals are at greater risk of developing certain diseases, such as lung disease, some cancers, cardiovascular diseases (CVD) [14, 16] and metabolic syndrome [17]. Additionally, cut-off thresholds cannot be used by gender and race universally, due to body composition differences [11, 18]. There are at least five proposed cut-off thresholds for different sexes, races and countries, considering the height effect over the metabolic risk in different populations [19].

Given these limitations, several authors have proposed the use of waist-to-height ratio (WHtR) as a better indicator of abdominal obesity. This measure corrects the WC according to the individual’s height and offers a strong correlation with cardiometabolic outcomes. Additionally, it is suggested that the main advantage of WHtR is its universal cut-off [13, 20]. A threshold value of 0.5 can be used for both genders, different ethnic groups and different age groups, thereby facilitating the diagnostic application of this tool and simplifying the public health message: "your waist circumference should be less than half of your height" [13, 21]. However, since the performance of the WHtR in detecting cardiometabolic risk factors has not been superior to the performance of BMI and WC in cross-sectional studies [22–24], and in a meta-analysis that included studies conducted with children and adolescents [25], its use is still controversy.

Therefore, the literature has not established the best anthropometric indicator to verify the association between obesity and HTN. A more suitable anthropometric method to detect individuals at increased cardiovascular risk is needed. In this regard, the present study aimed to evaluate and compare the discriminatory power of the WHtR in relation to the BMI and WC for the prediction of HTN in individuals from a small city in Brazil over a 13-year follow-up period.

## Methods

### Study population

The current study represents the second phase of a population-based observational prospective cohort study that was completed in 2015. The baseline cohort was initiated in 2002 in the city of Firminópolis, located in the midwest region of Brazil. At that time, the city had 9666 inhabitants, and the study included a representative sample of individuals aged over 18 years who were living in the urban area.

The sample size of the baseline cohort was calculated based on the total population at the time, a 25% prevalence of HTN, a 95% confidence interval and a 2,5% estimation error to obtain *n* = 1030. Twenty percent (20%) was added to this total to cover any losses (*n* = 1236). The sample comprised 1168 individuals (430 men and 738 women) [26].

The matrix design of the second phase, of which this study is one part, was approved by the Research Ethics Committee of the Clinical Hospital, Federal University of Goiás (Hospital das Clínicas da Universidade Federal de Goiás) (CEP/HC-UFG) with registration number 396,839. The study followed human research regulations according to National Health Council Resolution number 466/2012. The procedures were performed only after the participants signed the Terms of Free and Informed Consent.

This second phase included only individuals who did not have HTN at baseline. Thus, 400 individuals from phase 1 sample who had the disease at the time were initially excluded. Of the 768 remaining participants who were normotensive, 297 were excluded from phase 2. The reasons for exclusion were as follows: subject could not be found (*n* = 73), death (*n* = 55), moved to another city (*n* = 153), refusal (*n* = 5) and absent from their home (*n* = 11). The final study sample size was 471 (40,3%) individuals.

### Data collection

In 2015, all individuals found were interviewed in their own homes using a standardised questionnaire. All data were collected by a team of previously trained and constantly supervised researchers using standard equipment and techniques.

The questionnaire applied in this second phase was similar to the questionnaire used at baseline. In this study, the following information was analysed from each phase: Phase 1: identification and sociodemographic characteristics (age, gender, education, marital status), lifestyle (smoking, alcohol consumption, dietary intake and physical activity), self-reported health conditions (use of antihypertensive drugs) and objective measures (blood pressure, body weight, height and WC); and Phase 2: identification (only age and gender), self-reported health conditions (use of antihypertensive medications) and objective measures (only blood pressure).

### Anthropometric measurements

Anthropometric measurements were performed according to Lohman, Roche and Martorell’s recommendations [27]. Subjects were weighed and measured barefoot and wearing light clothing. Brand electronic scales with a precision of 100 g and a maximum capacity of 150 kg (Plenna model GIANT LITHIUM, São Paulo, Brazil) and a portable stadiometer with 0.1 cm precision (Seca model 206, São Paulo, Brazil) were used.

BMI was calculated dividing the weight (kg) by the square of height (m). Values ​​were classified as follows: < 18.5 kg/m^2^ (underweight), 18.5 to 24.9 kg/m^2^ (normal weight), 25 to 29.9 kg/m^2^ (overweight) and ≥30 kg/m^2^ (obese) for adults [28] and ≤22 kg/m^2^ (underweight), > 22 and < 27 kg/m^2^ (normal weight) and ≥27 kg/m^2^ (overweight) for the elderly [29]. Due to the small number of individuals in the “underweight” category (*n* = 32), this category was grouped together with the “normal weight” category.

The WC was measured with an inelastic tape measure positioned horizontally at the midpoint between the iliac crest and the last rib with the individual in a standing position and wearing minimal clothing. The measurement precision was 0.1 cm. The WC values ​​were classified and categorised as appropriate or increased (increased risk of cardiovascular disease) according to the cut-off points of < 94 cm and ≥94 for men and < 80 and ≥80 cm for women, respectively [10].

WHtR was calculated by dividing WC (cm) by height (cm), and.was categorised as follows: < 0.5 (appropriate) and ≥0.5 (increased: increased risk of cardiovascular disease) [20].

### Definition of hypertension (HTN)

A semi-automatic device (Omron HEM705CP, Kyoto, Japan) was used to measure blood pressure. The devices were periodically calibrated against a mercury column device to confirm their accuracy. Blood pressure classification and its measurement technique followed the recommendations of the VII Brazilian Society of Cardiology Arterial Hypertension Guidelines [30]. Two measurements were taken from the arm that had the highest initial pressure value. For analysis purposes, the mean value of the two measurements​ was used. The presence of HTN was defined as systolic blood pressure ≥ 140 mmHg and/or diastolic blood pressure ≥ 90 mmHg or the use of antihypertensive medications by the individual [30]; this measurement was considered the outcome variable in this second phase of the study.

### Confounders

The following variables were used as the confounders: gender, age (in years), education (complete years of study), marital status (resides with or without a partner), smoking (never smoked, smoker or ex-smoker), alcohol consumption (yes or no), physical activity (active or inactive) and food consumption (score I and score II).

To classify individuals as active or sedentary, information was collected concerning physical activities at work, commuting to work and during leisure time [31]. Individuals considered sedentary simultaneously in the three categories were classified as sedentary, and individuals considered active in at least one of the three categories were classified as active. Information concerning domestic activities was not included in the questionnaire. For more details about this classification refer to Additional file 1.

The individual’s food consumption was analysed using a food frequency questionnaire (FFQ) that estimated consumption during the previous year. The list of foods used was based on an FFQ developed and validated previously for a low-income adult population of the city of Goiânia, Brazil [32]. For the food consumption frequency to be treated as a quantitative variable, this measure was converted into a food consumption score that represented the individual’s daily intake in portions [33]. For each individual, the mean daily consumption frequency score of foods from the two food groups (Group A and Group B) was calculated. Group A was composed of all of the food contained in the FFQ that was associated with CVD risk, and Group B was composed of all of the food contained in the FFQ that was considered protective or not associated to CVD risk [30, 34, 35]. Refer to both groups food list on Table 1. Therefore, the “food consumption” variable was categorised into Score I and Score II, wherein the sum of the weighted values for foods in Group A was score I and the sum of the weighted values for foods in Group B was score II.

**Table 1:** Food groups according to risk of cardiovascular disease

Group A – Foods associated with cardiovascular disease	Group B – Foods not-associated with cardiovascular disease risk or protective
Milk and integral derivatives (Yogurt, curd, cheese and Creamy cheese)	Skimmed milk
Fat of animal origin (milk cream, butter, lard, Crackling, bacon)	Fat of Vegetable origin (soy oil, canola, corn and olive oil)
Fat of Vegetable origin (margarine and mayonnaise)	Fish in general (sardines, tuna, etc.)
Frying in general (egg, potato, meat, etc.)	Cereals, breads and tubers (rice, corn, potatoes, bread, flour, etc.)
Meats and sausages (beef, chicken, pork, viscera, sausage, sausage, mortadella, ham)	Legumes (beans, peas, lentils, etc.)
Canned Food (olive, tomato extract, heart of palm, etc.)	General vegetables (hardwood and non-hardwood)
Industrial drinks (soda and artificial juice)	Fruits in general and natural juices
Salts and preparations (fried and baked salted, package snacks, sandwiches, pizza, etc.)	Oleaginous (peanuts, chestnuts, almonds, hazelnuts, etc.)
Sugar and sweets in general	Whole foods (oat bran, wheat bran, brown rice, brown bread, etc.)

*CVD* cardiovascular diseases

### Statistical analysis

The statistical analysis was performed using the *STATA* program, version 12.0. Normality was tested using the Kolmogorov-Smirnov test. Student’s t-test for independent samples and Pearson’s Chi-square test were used to verify differences in the total sample in the first phase of the study (2002) according to the presence or absence of HTN in the second phase of the study (2015) among individuals.

BMI, WC and WHtR were tested separately using Poisson regression models by gender and adjusted for age, years of education, marital status, income, physical activity, smoking, alcohol consumption and food consumption. HTN was the outcome. The relative risk (RR) and its 95% confidence interval (CI) were obtained for each index assessed. Given the high correlation between the anthropometric indices analyzed (BMI/WC, *r* = 0.73; BMI/WHtR, *r* = 0.73; WC/WHtR, *r* = 0.90, *p* < 0.05), they were not considered independent variables in the same model.

The ROC (receiver operating characteristic) curves were analysed to identify the best cut-off points and to evaluate and compare the predictive capacity of the anthropometric indicators for the HTN outcome by age group in men and women (< 40 years of age and ≥40 years). The areas under the ROC curves provided the overall probability of the anthropometric indicator correctly classifying the presence or absence of SAH. A larger area under the ROC curve indicated a greater predictive power of the indicator. A perfect test has an area under the ROC curve equal to 1.0, whereas an area equal to 0.5 indicates that the test performance is no better than chance. Above 0.7 is considered a satisfactory performance. The lower 95% CI limit should not be less than or equal to 0.5 [36].

The sensitivity and specificity indicated the best cut-off points for the HTN risk. Sensitivity was defined as the ratio of correctly identified hypertensive individuals, and specificity was defined as the ratio of correctly identified normotensive individuals. The selected cut-off points had the greatest concomitant sensitivity and specificity [36].

The significance level adopted for all analyses was 5% with a confidence interval of 95%.

## Results

A total of 67.7% (*n* = 319) of the studied subjects (*n* = 471) were female. The mean follow-up period was 13.2 years. The total sample characteristics at baseline (2002) based on subsequent HTN development are shown in Table 2. Approximately 44.0% (*n* = 207) of the subjects developed HTN over the follow-up period. The subjects who developed HTN had a significantly higher age, lower education level, higher prevalence of smoking or being a former smoker and higher anthropometric measurements at the study baseline compared to the subjects who did not develop the disease (*p* < 0.05). Approximately 48.0% (*n* = 99), 54.0% (*n* = 112) and 73.0% (*n* = 150) (p < 0.05) of the subjects who developed HTN were overweight at baseline according to BMI, WC and WHtR, respectively.

**Table 2:** Characteristics at baseline (2002) of the total sample and the sample according to the subsequent development of hypertension

Variables	Overall (*n* = 471)	Hypertensives (*n* = 207)	Non-hypertensives (*n* = 264)
Gender, n (%)			
Male	152 (32.3)	74 (35.8)	78 (29.6)
Female	319 (67.7)	133 (64.2)	186 (70.4)
Age, years (mean ± SD)	38.9 ± 12.3	44.3 ± 12.2	34.7 ± 10.7*
Education, years (mean ± SD)	6.7 ± 4.0	5.9 ± 4.1	7.3 ± 3.8*
Marital status, n (%) (*n* = 466)			
With partner	338 (72.5)	141 (69.1)	197 (75.2)
Without partner	128 (27.5)	63 (30.9)	65 (24.8)
Physical activity, n (%)			
Active	270 (57.3)	116 (56.0)	154 (58.3)
Sedentary	201 (42.7)	91 (44.0)	110 (41.7)
Smoker, n (%)			
Never smoked	291 (61.8)	104 (50.2)	187 (70.8)*
Smoker	102 (21.7)	56 (27.1)	46 (17.4)
Ex-smoker	78 (16.6)	47 (22.7)	31 (11.7)
Alcohol consumption, n (%)			
Yes	170 (36.1)	75 (36.2)	95 (36.0)
No	301 (63.9)	135 (63.8)	169 (64.0)
Food consumption (mean ± SD)			
Score I	7.0 ± 2.3	6.91 ± 2.5	7.15 ± 2.2
Score II	10.5 ± 3.0	10.5 ± 3.2	10.5 ± 2.7
BMI, n (%)			
Normal weight	283 (60.1)	108 (52.2)	175 (66.3)*
Overweight	150 (31.8)	75 (36.2)	75 (28.4)
Obese	38 (8.1)	24 (11.6)	14 (5.3)
BMI, kg/m^2^ (mean ± SD)	24.5 ± 4.3	25.4 ± 4.6	23.7 ± 3.9*
WC, n (%)			
Appropriate	262 (55.6)	95 (45.9)	167 (65.3)*
Increased	209 (44.4)	112 (54.1)	97 (36.7)
WC, cm (mean ± SD)	82.7 ± 10.3	85.7 ± 10.5	80.2 ± 9.5*
WHtR, n (%)			
Appropriate	187 (39.7)	57 (27.5)	130 (49.2)*
Increased	284 (60.3)	150 (72.5)	134 (50.8)
WHtR (mean ± SD)	0.52 ± 0.07	0.54 ± 0.07	0.50 ± 0.06*

*SD* Standard deviation, Score I: Daily intake of foods considered to increase risk of cardiovascular diseases (CVD); Score II: Daily intake of foods considered protective of or having no effect on risk of *CVD*; *BMI*: Body mass index; *WC*: Waist circumference; WHtR: Waist-to-height ratio

*Significant difference between variables according to subsequent development of hypertension (*p* < 0.05)

Firminópolis, Brazil (2002–2015)

Table 3 shows the relative risk (RRs) and 95% confidence intervals for the associations between the anthropometric measurements and cumulative incidence of HTN after 13 years by gender. All anthropometric indices were associated with the risk of developing HTN among women after adjusting for age, education, marital status, income, physical activity, smoking, alcohol consumption and food consumption. The risk of new HTN cases was 82.0% for obese individuals (RR: 1.82; 95% CI: 1.07 to 3.09), 71.0% for an increased WC (RR: 1.71; 95% CI: 1.15 to 2.54) and 66.0% for an increased WHtR (RR: 1.66; 95% CI: 1.08 to 2.56) in women. The associations were not significant for men after adjustment (*p* > 0.05).

**Table 3:** Adjusted relative risk (RR) and 95% confidence interval for the risk of developing hypertension according to the anthropometric measurement categories obtained at baseline (2002) by gender

Variables	Overall (*n* = 471)		Men (*n* = 152)		Women (*n* = 319)	
	Number	RR (95% CI)^a^	Number	RR (95% CI)^b^	Number	RR (95% CI)^b^
BMI						
Normal weight	283	1.00	79	1.00	204	1.00
Overweight	150	1.29 (0.96–1.74)	61	1.05 (0.63–1.74)	89	1.44 (0.98–2.11)
Obese	38	1.40 (0.90–2.18)	12	0.91 (0.37–2.26)	26	1.82 (1.07–3.09)*
WC						
Appropriate	262	1.00	120	1.00	142	1.00
Increased	209	1.46 (1.08–1.98)*	32	0.87 (0.49–1.55)	177	1.71 (1.15–2.54)*
WHtR						
Appropriate	187	1.00	68	1.00	119	1.00
Increased	284	1.80 (1.33–2.44)*	84	1.29 (0.78–2.15)	200	1.66 (1.08–2.56)*

*CI* Confidence interval, *BMI* Body mass index, *WC* Waist circumference, *WHtR* Waist-to-height ratio

^a^Estimated using Poisson regression model adjusted for gender, age, education, marital status, income, physical activity, smoking, alcohol consumption and food consumption

^b^Estimated using Poisson regression model adjusted for age, education, marital status, income, physical activity, smoking, alcohol consumption and food consumption

*p < 0.05

Firminópolis, Brazil (2002–2015)

**Fig. 1:**
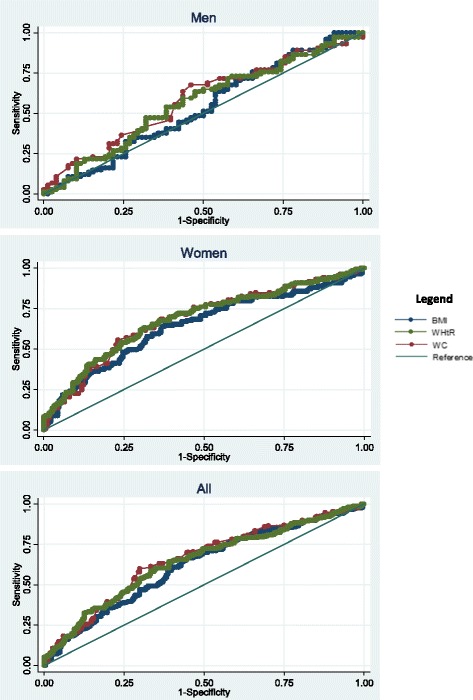
Area under the receiver operating characteristic (ROC) curve (AUC) for the body mass index (BMI), waist circumference (WC) and waist-to-height ratio (WHtR) in the prediction of hypertension for the total sample and by gender. Firminópolis, Brazil (2002–2015)

Table 4 shows the areas under the ROC curve (AUC) of the anthropometric measurements for HTN prediction. All anthropometric indices showed a reasonable predictive capacity in detecting the presence of HTN in women. However, this capacity occurred at random for men (95% CI < 0.5). The discriminatory power of the indices for HTN in women was approximately 64.0% for BMI (AUC: 0.64; 95% CI: 0.58–0.71), 68.0% for WC (AUC: 0.68; 95% CI: 0.62–0.74) and 69.0% for WHtR (AUC: 0.69; 95% CI: 0.63–0.75). The AUC values ​​did not differ significantly between BMI, WC and WHtR in men and women in either the total sample analysis or the age-stratified analysis. The AUC for the WHtR was significantly higher than the AUC for BMI among women only in the paired comparison, however, the differences between the AUCs were small (0.05). The ROC curves for the total sample and by gender are shown in Fig. 1.

**Table 4:** Area under the receiver operating characteristic (ROC) curve (AUC) and 95% confidence interval for the prediction of hypertension according to anthropometric measurements obtained at baseline (2002) by age group in men and women

Age group	Number	AUC (95% CI)			*p* for all*	*p* for comparison in pairs*		
		BMI	WC	WHtR		BMI vs.WC	BMI vs. WHtR	WC vs. WHtR
Men								
< 40	86	0.56 (0.43–0.69)	0.62 (0.49–0.74)	0.59 (0.46–0.72)	0.358	0.348	0.575	0.170
≥ 40	66	0.57 (0.42–0.73)	0.54 (0.39–0.68)	0.50 (0.34–0.64)	0.158	0.648	0.294	0.091
Total	152	0.53 (0.44–0.63)	0.59 (0.49–0.68)	0.57 (0.48–0.66)	0.305	0.232	0.422	0.219
Women								
< 40	197	0.63 (0.54–0.73)	0.65 (0.56–0.73)	0.62 (0.53–0.71)	0.214	0.682	0.555	0.080
≥ 40	122	0.61 (0.50–0.71)	0.64 (0.53–0.75)	0.65 (0.55–0.75)	0.553	0.352	0.276	0.567
Total	319	0.64 (0.58–0.71)	0.68 (0.62–0.74)	0.69 (0.63–0.75)	0.130	0.057	0.045	0.780
Total sample	471	0.62 (0.57–0.67)	0.66 (0.61–0.71)	0.64 (0.59–0.69)	0.125	0.064	0.271	0.225

*AUC* Area under the curve, *CI* Confidence interval, *BMI* Body mass index, *WC* Waist circumference, *WHtR* Waist-to-height ratio, vs. versus. *p Value of difference between AUC (*p* < 0.05)

Firminópolis, Brazil (2002–2015)

The cut-off points of the three anthropometric indices with higher HTN predictive capacities using the ROC curve analysis are summarised in Table 5. For men, the optimal cut-off points for predicting HTN were 24.8 kg/m^2^ for BMI, 85.3 cm for WC and 0.50 for WHtR. For women, the optimal cut-off points were 23.8 kg/m^2^ for BMI, 81.8 cm for WC and 0.52 for WHtR. The cut-off values did not significantly vary with age, although there was a slight increase among individuals under 40 years of age.

**Table 5:** Cut-off points for anthropometric measurements in the prediction of hypertension according to age group in men and women

Age group	Number	BMI			WC			WHtR		
		Cut-off point (km/m^2^)	S (%)	Sp (%)	Cut-off point (cm)	S (%)	Sp (%)	Cut-off point	S (%)	Sp (%)
Men										
< 40	141	25.64	63.6	57.7	87.00	63.6	69.2	0.501	63.6	65.4
≥ 40	11	24.80	55.0	41.5	87.00	55.0	55.2	0.501	55.0	51.7
Total	152	24.80	51.4	50.0	85.30	63.5	56.4	0.503	59.5	56.4
Women										
< 40	293	23.89	66.7	67.1	81.00	66.7	65.8	0.507	73.3	63.0
≥ 40	26	23.82	61.5	64.3	79.00	66.7	61.4	0.506	59.0	54.3
Total	319	23.82	63.9	62.9	81.50	63.2	66.7	0.523	66.2	64.5

*BMI* Body mass index, *WC* Waist circumference, *WHtR* Waist-to-height ratio, *S* Sensitivity, *Sp* Specificity

Firminópolis, Brazil (2002–2015)

## Discussion

The results indicate that overweight women are at a greater risk of developing HTN. The anthropometric body fat indices (total and abdominal) were associated with a risk of developing HTN over the 13 years of follow up, which corroborated the results of other studies that found that excess fat was a major determinant of HTN development [9, 37, 38].

Risk estimates from population studies show that approximately 75% of diagnoses of HTN may be attributed directly to obesity [3, 4]. The mechanisms involved in obesity-induced HTN are complex and need to be further understood. Some authors have noted several physiological changes that occur in people with excess body fat, such as activation of the sympathetic nervous system and the renin-angiotensin aldosterone system, endothelial dysfunction and/or functional abnormalities, may result in hypertension [39, 40].

In this study, the proportional hazards regression analysis adjusted for the confounders showed a direct association between BMI, WC and WHtR and the risk of HTN, but only for women. These anthropometric indicators have also been associated with HTN in longitudinal [8, 41, 42] and cross-sectional [7, 23, 43–46] studies in different populations. In a study conducted in public hospitals in Argentina, BMI, WC and WHtR predicted HTN incidence after 10 years of follow-up. The hazard ratio was higher for WC and WHtR when compared to BMI [8]. In Brazil, all anthropometric indices assessed (including WC and WHtR) were significantly associated with HTN incidence with the exception of BMI in a cohort of adults living in the south of the country after a mean follow up of 5.6 years [42]. This finding contrasts with the findings of the present study, in which all three anthropometric indices showed similar predictive capacities in women.

In a population-based cross-sectional study in large Brazilian cities, Peixoto et al. [44] observed that HTN was equally associated with an increased WC in adults from both genders. An association between BMI and SAH was found only for women. Silva et al. [23] found that a high BMI, WC and WHtR were associated with new HTN cases in adults of both genders. WC was the best independent predictor of HTN for women, whereas BMI was the best predictor for men. A high WHtR was associated with SAH only in women, whereas BMI was the indicator most strongly associated with the disease in both genders [7].

One possible reason for the differences in associations between anthropometric indices and HTN risk in such studies is that these figures may not reflect the same risk in different populations. Differences in body composition and fat distribution between different ethnic groups, genders and age groups have been well discussed in the literature [11, 47, 48]. Additionally, the study design and variations in the type of statistical analysis used or the choice of adjustment variables can modify the degree of association between anthropometric indices and HTN. For example, in our study, the main variables related to increased HTN onset were adjusted. For the total sample (*n* = 471), a significant association was found between abdominal obesity indices and HTN risk. However, when the analysis was stratified by gender, all obesity indices lost significance in the associations in relation to men. This result showed that the indicators were associated with the risk of disease only in women.

Corroborating our results, Chei et al. [41] observed that the assessed anthropometric measurements were not significantly associated with HTN risk in men in a Japanese cohort after analysis by gender adjusted for confounders. Therefore, studies conducting risk analyses only for the total sample and including “gender” only as a covariate in the adjusted model may not reflect the real association between anthropometric indices and the risk of developing the disease [8, 42, 43, 46].

In contrast to earlier studies [6, 13, 20, 49], no significant difference between anthropometric indices was observed in our population for HTN prediction HTN according to gender and age when analysed using areas under the ROC curve. The results showed that the BMI, WC and WHtR had a similar likelihood of classifying the presence or absence of HTN in women. Oppositely, the indices were not capable of predicting the disease among men. This difference may be explained by the smaller number of males in the sample.

Notably, the accuracy of anthropometric variables as a predictor of HTN was not high in this study, since it has been reported [50] that an AUC between 0.5 and 0.7 indicate that the predictive capacity is less precise. A similar result was found in a recent study in the United States with adult women, which showed that the three anthropometric measurements had similar modest capacities for HTN prediction (AUC = WC: 0.63; WHtR: 0.65 and BMI: 0.66) [51].

In previous studies with a cross-sectional design, the AUC values ​​did not differ between BMI, WC and WHtR in adults in China [22, 52] and Brazil [23] for either gender in HTN prediction. However, in a study of Spanish elderly people at high cardiovascular risk, the AUC for BMI was significantly higher than the AUCs for WC and WHtR in a model adjusted for confounders, although the magnitude of the differences was considered small (AUC: 0.67, 0.66 and 0.66, respectively) [49].

In a study of 36,642 adults (both men and women) in China, the AUC for the WHtR was significantly greater than the AUC of BMI or WC in HTN prediction [6]. The authors also observed that the WHtR was able to predict cardiometabolic risks, including HTN, among individuals with an appropriate BMI and WC. Similar results were found in a large study in Japan (*n* = 45,618). In an unadjusted model, the AUCs for WHtR were also significantly greater than the AUCs for BMI and WC in both genders. However, no differences were observed after adjusting for age [24]. Systematic review and meta-analyses also indicated that WHtR might be the best anthropometric index to predict a broad range of cardiometabolic risk factors associated with central obesity, including HTN [20, 13].

The results of our study showed that the correction of the WC according to the individual’s height reduced the magnitude of the association with the incidence of HTN in the risk analysis. Furthermore, comparison of the AUCs revealed no difference in the prediction of the analysed indices. This finding suggests that either the overall obesity (BMI) or central obesity (WC and WHtR) anthropometric indicators can be used in Brazilian adults to evaluate the risk of HTN, as suggested in another cross-sectional study conducted in Brazil [23].

The cut-off points that showed the best predictive capacity for the HTN risk in the present study based on sensitivity and specificity were similar to the currently recommended values (25 kg/m^2^ and 80 cm) [10, 28] for excess weight according to BMI (both genders) and increased WC (in women), respectively. The cut-off point with a higher variation was increased WC among men (85.3 cm), which was 8.7 cm lower than the current recommendation (94 cm) [10]. Similar results were found in a study in southern Brazil [23]. The best cut-off values ​​for BMI associated with HTN risk were also close to the current recommendations (24.9 and 24.6 kg/m^2^ for women and men, respectively). For WC, the cut-off point was higher for women (86.2 cm) than for men (89.5 cm) considering the current recommendations for an increased WC.

In a previous study in the midwest region of Brazil [44], the cut-off points for BMI and WC, which offered the best predictive capacity for the risk of HTN, were identical to the currently recommended values ​​for excess weight and increased WC in women. For men, the cut-off point for an increased WC (86 cm) was well below the recommended values, as was the case for the men in this study. The difference found between the cut-off for WC and the value recommended by the literature suggests that some men in this study were classified incorrectly regarding the HTN risk, which may generate a high number of false negatives.

In relation to the WHtR, the cut-off values ​​found in this study for both genders and for all age groups were similar to the value currently recommended in the literature for different populations (0.5) [6, 13, 21, 23]. The similarities found between the WHtR cut-off points underscore the fact that the WC of a particular person should not be more than half of the person’s height.

Some limitations of the present study merit attention. First, changes in obesity indicators during follow-up were not considered in the analyses and might have affected the results. Additionally, only the prediction of HTN risk was evaluated in this study. Other diseases associated with obesity, such as dyslipidaemia and diabetes, were not included due to the lack of data for these analyses.

Additionally, we could not monitor all individuals in the second phase of the study due to losses and exclusions. However, this limitation probably did not affect the results, because if the subjects evaluated in phase 2 (*n* = 471) were compared to those not evaluated (*n* = 297), no differences were found in the general characteristics of the groups (*p* > 0.05).

The strength of this study is its longitudinal design, which allows evaluation of exposure-outcome temporality. Notably, the anthropometric and blood pressure measurements were collected according to standard protocols by trained researchers. Finally, because hypertension is a disease of multifactorial origin, the risk analysis performed considered the main disease risk factors for adjustment, including food consumption. This consideration was not observed in most similar studies, which differentiated this study from others.

## Conclusions

In conclusion, this study found that high BMI, WC and WHtR values were associated with increased risk of developing HTN among women and had similar disease-predicting capacities. The cut-off points of the analysed indices were similar to the current recommendations, except for the WC for men. These results reinforce the importance of using anthropometric indices as part of the public health programs strategy. It would help preventing and controlling overweight epidemic as well as warn about the risk of developing HTN and other comorbidities associated to weight gain. Although WHtR was not a superior disease-predictor, it can be used as an effective and simple toll to identify individuals at risk of developing HTN.

## Additional file

Additional file 1: Physical activity classification. Criteria used to classify individuals as active or sedentary according to physical activity. (DOCX 12 kb)

## Abbreviations

AUC: Area under the curve
BMI: body mass index
*CI*: Confidence Interval
CVD: cardiovascular diseases
FFQ: food frequency questionnaire
HTN: hypertension
ROC: receiver operating characteristic
RR: relative risk
SAH: systemic arterial hypertension
WC: waist circumference
WHtR: waist-to-height ratio
