# Supplementary material for: Is waist-to-height ratio the best predictive indicator of hypertension incidence? A cohort study
Source: BMC Public Health. 2018 Feb 26;18:281. doi: 10.1186/s12889-018-5177-3 (PMC6389116; doi:10.1186/s12889-018-5177-3)
Supplement: Supplementary file 1 — Physical activity classification. Criteria used to classify individuals as active or sedentary according to physical activity. (DOCX 12 kb) [file 12889_2018_5177_MOESM1_ESM.docx]

**ADDITIONAL FILE 1**

**Physical activity classification**

To classify individuals as active or sedentary, information was collected concerning physical activities at work, commuting to work and during leisure time.

Individuals considered sedentary simultaneously in the three categories were classified as sedentary, and individuals considered active in at least one of the three categories were classified as active. Information concerning domestic activities was not included in the questionnaire.

Leisure time physical activities: sedentary (when most part of the leisure time was spent with activities that resulted in low energy expenditure, such as watching TV, using the computer etc.); mild physical activity (when some part of the leisure time was spent with activities such as riding a bicycle, running or practicing sports), moderate physical activity (when most part of the leisure time was spent with activities such as riding a bicycle, running or practicing sports) and vigorous physical activity (when an athlete was identified, whose free time was used in trainings for competitions, running or other sports).

Commuting physical activity – individuals were asked if they walked or ride a bike to get to work, and how long did they spend in those activities daily. Then they were classified as sedentary (<15 minutes of physical activity to get to work) or active (≥ 15 minutes of physical activity to get to work).

Physical activities at work: sedentary (spent most of the time sitting or performed activities that involved little physical exertion); mild physical activity (little walking while working, with no possibility of lifting or carrying heavy objects), moderate physical activity (walked a lot while working, with the possibility of lifting or carrying heavy objects) and vigorous physical activity (performed extenuating work, or had to carry heavy objects).
